# Editorial: Artemisinin—From Traditional Chinese Medicine to Artemisinin Combination Therapies; Four Decades of Research on the Biochemistry, Physiology, and Breeding of *Artemisia annua*

**DOI:** 10.3389/fpls.2020.594565

**Published:** 2020-09-18

**Authors:** Tomasz Czechowski, Pamela J. Weathers, Peter E. Brodelius, Geoffrey D. Brown, Ian A. Graham

**Affiliations:** ^1^ Centre for Novel Agricultural Products, Department of Biology, University of York, York, United Kingdom; ^2^ Department of Biology and Biotechnology, Worcester Polytechnic Institute, Worcester, MA, United States; ^3^ Department of Chemistry and Biomedical Sciences, Linnaeus University, Kalmar, Sweden; ^4^ Department of Chemistry, University of Reading, Reading, United Kingdom

**Keywords:** *Artemisia annua*, artemisinin, semi-synthetics, molecular breeding, malaria

The 2015 Nobel Prize in Physiology or Medicine was awarded to Tu Youyou for her “discoveries concerning a novel therapy against malaria”. Educated in pharmaceutical sciences, Tu was recruited to Chinese military research Program 523, with the aim of finding new drugs for the treatment of malaria. A malaria epidemic during the Vietnam War had led Ho Chí Minh, the Prime Minister of North Vietnam, to request medical help from China. In response, Chairman Mao approved Project 523, which involved over 500 scientists, military personnel, and medical practitioners and ran from 1967 to 1980. Whilst reviewing written records of traditional Chinese medicine, Tu noticed a mention of Qinghao (*Artemisia annua*) for alleviation of malaria fevers in Ge Hong’s “A handbook of prescriptions for emergencies”, which has been dated to around 317–420 A.D. She next found that an ethyl ether extract from *A. annua* leaves strongly inhibited malaria, leading Tu and two other members of her team to test the Qinghao plant extract for safety and side-effects on themselves. In 1972, Tu´s team obtained the pure active substance from this extract and determined its chemical structure, naming it as qinghaosu, or artemisinin, as it became more commonly known in the West. A series of chemical derivatives of artemisinin were subsequently developed by Project 523, including dihydroartemisinin, artemether, and artesunate. These compounds have become part of the artemisinin combination therapies (ACTs), currently the World Health Organisation (WHO)-recommended first-line drugs to combat malaria.

Almost fifty years after Tu´s discovery, malaria still poses a global threat, with an estimated 228 million cases occurring worldwide in 2018 causing 405,000 deaths – two thirds of them among children under 5 years old in sub-Saharan Africa (World malaria report, 2019). The introduction of ACTs (it is estimated that 3 billion treatment courses have been procured worldwide between 2010 and 2018), rapid diagnostic tests (RDTs) and malaria vector controls, including insecticide-treated mosquito nets, reduced the number of cases significantly over the past 10 years (World malaria report, 2019). However, artemisinin resistance conferred by genetic mutations in *Plasmodium falciparum* recently emerged in the Greater Mekong sub-region (Ariey et al., 2014) together with *Pfhrp2/3* gene deletions that render parasites undetectable by RDT (Owusu et al., 2018), represent major new threats in the global fight against malaria.

*A. annua* remains the sole global source of the drug at the time of writing, despite significant efforts to develop alternative production platforms, as discussed herein. National malaria programmes delivered 214 million ACT treatment courses in 2018 (WHO Malaria report, 2019), equating to around 100 metric tonnes of pure artemisinin obtained from *A. annua* (assuming 0.5 g artemisinin per treatment). The plant-sourced drug demand stimulated breeding efforts to improve yields. Widely practiced phenotypic selection of open pollination varieties has been supplemented by modern molecular breeding approaches, resulting in *A. annua* F1 hybrids that yield almost 55 kg of artemisinin per hectare with a content reaching 1.44% of leaf dry weight (Artemisia F1 Seed). Several publications herein suggest that natural variation within *A. annua* populations may have even more to offer in terms of further improving yields. Wetzstein et al. show that further yield improvements can be achieved through the use of phenotypic-based selection and clonally propagating high-yielding genotypes. Work from Ferreira et al. shows the importance of a thorough understanding of seasonal variation of artemisinin content in the *A. annua* crop when planning harvest and maximising artemisinin returns from plantations. That work also highlights existing differences in response to photoperiod among different chemotypes of *A. annua.* Czechowski et al. shed new light on the molecular basis of the existence of high- and low- artemisinin producing chemotypes, providing candidate targets for yield improvement through molecular breeding.

Transgenic routes, also being explored for further artemisinin yield improvement in *A. annua*, have resulted in the elucidation of much of the artemisinin biosynthetic pathway and the identification of multiple transcriptional regulators of biosynthetic genes, as reviewed by Ikram and Simonsen The same work also reviews transgenic approaches that have succeeded in achieving an artemisinin content of 2.6% leaf dry weight by overexpressing biosynthetic genes; and over 3%, when metabolic pathways competing for the five-carbon isoprenoid precursors are blocked. Ma et al. and Tang et al. report on further transgenic approaches that identify additional genes involved in the regulation of artemisinin biosynthesis in *A. annua.* Fu et al. present work adding to the sparse knowledge of the transport systems potentially involved in regulation of artemisinin biosynthesis, which may prove to be a valuable alternate route for genetic improvement of artemisinin production. A number of the transgenic approaches to improve artemisinin yield appear to come at the cost of plant biomass and fitness, highlighting the need for extensive field trials to validate laboratory and glasshouse data. Regulatory approval will be required before release of these GMOs, which remains a challenge for this high-profile medicinal plant. Transgenics is, of course, also an extremely valuable experimental tool for characterisation of *in planta* gene function, yielding knowledge that is useful for both genome editing and molecular breeding. In this context, Zhang et al. report on the characterisation of genes involved in the supply of isoprene precursors from the MEP-pathway and Catania et al. report on the effects of silencing the first committed step in artemisinin biosynthesis using an RNAi approach. This latter study also opens up the prospect of using *A. annua* as a production platform for other high value sesquiterpenes.

Attempts to transfer artemisinin production to other plant and microbial hosts are reviewed by Ikram and Simonsen and Kung et al. Achievements of the Keasling group in engineering *Saccharomyces cerevisiae* that produces the artemisinin precursor, artemisinic acid, at yields of 25 g/L remains an exemplar for successful metabolic engineering (Paddon et al., 2013). However, costs associated with the non-enzymatic photochemical conversion of artemisinic acid to artemisinin have proved too expensive to allow the semi-synthetic route to compete with plant-based production, where both enzymatic and non-enzymatic steps are conducted in glandular secretory trichomes, which are specialized 10-cell structures found on the surface of the leaf, stems and flower buds (Peplow, 2016). Kung et al. discuss recent developments in the chemical conversions of artemisinic acid to artemisinin potentially replacing costly photochemical processes developed by Sanofi, raising the prospect once again of an alternative source of artemisinin that would help stabilise supply.

Ikram and Simonsen review the prospect of engineering plant heterologous systems for artemisinin production and report a proof-of-concept in *Nicotiana* species, albeit at yields significantly less than for *A. annua* itself. The presence of endogenous glycosyltransferases in *Nicotiana* species that are able to glycosylate the engineered artemisinin precursors, rendering them unsuitable for further spontaneous conversions into artemisinin, makes the use of these species as production hosts particularly challenging. In an effort to find an alternative host with less glycosyltransferase activity, Ikram et al. have evaluated the non-vascular plant, *Physcomitrella patens.* The artemisinin levels achieved in this moss species were significantly higher than those from *Nicotania* species, but still around 100-times lower than those found in *A. annua*.

The use of monotherapies that rely on artemisinin or its derivatives as the sole antimalarial agents is not recommended by the World Health Organisation since this practice significantly increases the risk of the emergence of parasite resistance. Some previous literature has supported the use of *A. annua* herbal remedies as cost-effective alternatives to ACTs with the suggestion that artemisinin works in combination with other compounds, such as flavonoids (for example Weathers et al., 2014). Czechowski et al. used *in vitro* assays with whole plant extracts from a series of *A. annua* mutants, deficient in either the production of artemisinin or flavonoids, to demonstrate that artemisinin is the sole metabolite from *A. annua* with *in vitro* anti-plasmodial activity. The possibility of other compounds having *in vivo* effects was also discussed as it is recognised that *in vitro* and *in vivo* studies do not always recapitulate one another in therapeutics development. This study has recently been cited as evidence in a WHO position paper that does not support the promotion or use of Artemisia plant material in any form for the prevention or treatment of malaria (WHO, 2019).

## Author Contributions

All authors contributed to the production of the Research Topic and/or the editorial.

## Conflict of Interest

The authors declare that the research was conducted in the absence of any commercial or financial relationships that could be construed as a potential conflict of interest.
